# Coverage with Evidence Development Programs for Medical Technologies in Asia-Pacific Regions: A Case Study of Japan and South Korea

**DOI:** 10.31662/jmaj.2021-0011

**Published:** 2021-09-13

**Authors:** Jae-Eun Myung, Yuji Tanaka, Hyunsook Choi, Liesl Strachan, Tomohiro Watanuki, Ji-Hyun Lee, Hyojung Hwang, Sang-Soo Lee

**Affiliations:** 1 Healthcare Economics and Government Affairs, Medtronic Korea Ltd., Seoul, South Korea; 2 Healthcare Economics and Government Affairs, Medtronic Japan Ltd., Tokyo, Japan; 3 Department of Health Convergence, Ewha Womans University, Seoul, South Korea; 4 Global Health Policy, Medtronic Australasia Pty Ltd., Sydney, Australia; 5 Department of Health Policy, Graduate School of Public Health, Yonsei University, Seoul, South Korea; 6 Graduate School for Medical Device Management and Research, SAIHST (Samsung Advanced Institute for Health Science & Technology), Sung Kyun Kwan University, Seoul, South Korea

**Keywords:** coverage with evidence development, CED, Challenge Application, Conditional Selective Benefit, medical technology, policy, Japan, South Korea

## Abstract

In this article, the operational characteristics of coverage with evidence development (CED) programs in Asia-Pacific regions, focusing on two countries―Japan and South Korea―are reviewed. Both countries recommended the introduction of CED to overcome the barrier of lack of robust clinical evidence in the early stages of the introduction of a medical technology. However, each country has a unique approach to CED implementation that reflects the differences in establishment and healthcare and policy environments. Japan adopted a “Challenge Application (CA)” program in 2018, and South Korea introduced the “Conditional Selective Benefit (CSB)” program in 2014. Despite the positive effects of CED programs, their governance and implementation should be improved to benefit patients in both countries from the improved access to new and innovative medical technologies. To this end, CED practices in the United States (the USA) can provide insights on how to improve CED operations in both countries.

## Introduction

While new innovative medical technologies have substantially contributed in saving lives and improving the health outcomes of patients, governments and payers around the world continue to face the challenge of providing access to these technologies while ensuring healthcare expenditure and budgets remain sustainable. Indeed, even in countries where the evaluations of the health benefits of a new technology are undertaken to justify its additional costs, such as cost-effectiveness analyses or value-for-money assessments, decisions on access and funding remain contentious, particularly where uncertainties about clinical effectiveness and value exist ^(1)^. In an effort to provide patient access to new innovative medical technologies while managing their budgetary impact and use, a range of alternative payment models, known as Managed Entry Agreements (MEAs), has been proposed ^(2)^. MEAs are not new; indeed, the pharmaceutical sector have actively pursued these arrangements with payers for decades to provide access to medicines where uncertainties exist ^(3), (4)^. In general, MEAs are largely composed of finance-based agreements, such as price-volume agreements, and performance-based agreements ^(5), (6)^, which includes risk-sharing and payment upon achieving certain outcomes. Coverage with evidence development (CED) programs are an example of one population-level performance-based MEA^(7)^.

In the early stages of medical technology commercialization, a lack of sufficient and robust evidence results in uncertainties around the clinical and cost-effectiveness of the technology. As a result of such uncertainties, coverage determination procedures may be challenged, limiting access to patients in need. CED initiatives have been introduced to help answer uncertainties around costs and outcomes by generating evidence on the impact and utilization of new medical technologies ^(8), (9)^. CED, which is defined as a conditional coverage and payment program whereby temporary or interim funding and access to new medical technologies is granted under the condition that evidence is collected in parallel to answer the uncertainties surrounding their clinical and/or economic value, has been introduced to overcome these limitations ^(9)^.

Nondrug medical technologies, such as implantable or disposable medical devices, surgical equipment, *in vitro* diagnostics, and even wearable devices, are fundamentally different from pharmaceuticals in ways that have been well documented ^(10), (11), (12)^. Some of these differences include but are not limited to the speed and incremental nature of device innovation, device-operator interactions, and the learning curve effect and licensing or regulatory requirements. Of course, many of these key differences translate into differences in the amount and type of evidence that can be produced to demonstrate the safety and effectiveness of nondrug technologies, particularly the ability to conduct double-blinded randomized studies due to recruitment issues and ethical challenges ^(13)^. This lack of evidence leads to challenges for those undertaking health technology assessment (HTA) or making coverage decision. In Korea, if no clinical study evidence pertaining to a medical technology exists, it cannot even be the subject of HTA review.

As a mechanism to address the challenges confronting medical technologies, decision-makers and manufacturers have used CED programs to fill the gap of required evidence such as safety and effectiveness in the early market entry stage of medical technologies. In the United States (the USA), the concept of CED emerged over a decade ago when the Centers for Medicare and Medicaid Services (CMS) introduced the CED program in 2006^(14)^. Since then, the CMS CED program has evolved with the recent successful inclusion of transcatheter aortic valve replacement (TAVR) technologies and leadless pacemakers in this CED program ^(9), (15)^. Other countries such as France ^(16), (17)^ and Sweden ^(18), (19)^ are also actively undertaking CED programs in various forms, demonstrating the usefulness of CED as an important tool to secure access to new drugs and medical technologies ^(20)^.

In this study, the authors examined the operational characteristics of CED programs in Asia-Pacific regions, focusing on two countries ―Japan and South Korea (hereafter Korea)―both of which have similar universal healthcare systems and mechanisms to reimburse new medical technologies.

## Coverage with Evidence Development Program in Japan and Korea

Both Japan and Korea have universal social health insurance systems and numerous similarities in the way medical technologies are paid for, such as fee-for-service, limited operation of prospective payment systems known as diagnosis-related groups, and combined fee schedules for hospital and physician fees. Both countries have a functional category payment system for managing medical technologies, including a stand-alone payment for implantable or high-cost disposable devices, low-cost disposable devices and instruments paid under procedure fees, and a premium pricing rule for innovative new medical technologies by creating new functional categories. However, some differences exist between the two countries, for instance, how premium prices are determined (Table 1).

**Table 1.** The Reimbursement and Pricing Categories for Medical Technologies in Japan and South Korea ^(41), (42)^.

Category		South Korea	Japan
Coverage	Reimbursed	Comparing the technologies with those already listed and placed in the same “functional category,” which has similarity in material, feature, size, etc.	B1 category: placed in the existing functional classification
			B2 category: placed in the existing functional classification with definition change
			B3 category: placed in the existing category with improvement premium with conditional period
		If the new technology is considered superior to the listed technologies, “Value Appraisal Standard” Innovation Track and Technology Improvement Track are applied for premium pricing and a new functional category is created	C1 category: premium pricing with new functional category creation
			C2 category: new functional category with new procedure code or only new procedure code
			R category: evaluation of remanufactured technologies (reprocessed technologies with new functional category)
	Funded under the procedure (technical) fee (integrated fee schedule of hospital and physician fees)	Low-cost disposable and reusable instruments are paid under procedure fees	A1 category: placed in the existing technical fees that are not linked to the specific procedure fee codes
			A2 category: placed in the existing technical fees that are linked to the specific procedure fee code
			A3 category: placed in the existing procedure fees, but change the condition of the fees
Noncoverage		Cosmetic, not cost-effective or not clinically essential technologies	F category: Not suitable for reimbursement coverage

Both countries adopted CED programs suitable for their own healthcare environment. Japan adopted the “Challenge Application (CA)” program, and Korea introduced the “Conditional Selective Benefit (CSB)” program. In April 2018, the Ministry of Health, Labour and Welfare (MHLW) of Japan released revised notifications outlining reimbursement schemes for medical technologies of which the CA program was newly established as one of the major revisions. The program was developed to address the difficulties of verifying the final assessment of coverage determinations for long-term implantable technologies or highly innovative medical technologies before they are listed for coverage and reimbursement. Under the CA program, medical technologies that lack appropriate clinical evidence at the initial listing stage are eligible for reevaluation after evidence has been generated. It provides an opportunity of earlier patient access and premium prices by proving clinical value with additional evidence ^(21)^.

In Korea, the Ministry of Health and Welfare (MoHW) introduced the CSB program in 2014 to provide preliminary benefits for services and items that required additional evidence to demonstrate its safety, effectiveness, or cost-effectiveness but has potential benefit to patient care. Before the introduction of the program, the services and items under the CSB program were generally determined as noncovered services or items resulting in out-of-pocket costs and often a large financial burden to patients.

## “Challenge Application” Program in Japan

The CA program evaluates clinical evidence generated during the post-market period. It was initiated to provide an opportunity for medical technology manufacturers to apply for the reevaluation of their technologies based on evidence that will be generated after the initial listing of the eligible technologies for coverage and reimbursement. The program was created as a response to a request from the medical technology industry. The development of the CA program involved detailed discussions in an expert panel of Special Treatment Materials (STMs, medical technologies listed in a specific functional category, such as coronary stent and pacemaker). Findings and recommendations from the expert panel are then followed by an approval from the “Central Social Insurance Medical Council (CSIMC, called Chuikyo).” The CSIMC is an advisory body of MHLW, which deliberate over revisions of the health insurance system and medical fees ^(22)^. The expert panel reports to the CSIMC, and it assesses and deliberates the reimbursement application of STMs, including the CA program ^(23)^. Manufacturers applying for new STMs can request to be included in the CA program if their application falls into the following group: B1, B2, B3, C1, and C2. In the case of C2, STMs are only subject to CA program, and the details of each category are described in Table 1.

Not all categories were subject to CA program in the beginning. Initially, the target of CA was limited to B3, C1, and C2 categories. After 2 years, the target was extended to B1 and B2 categories. Products listed before the introduction of the CA program were given the opportunity to apply for the program for certain period. B3, C1, and C2 categories listed before the initiation of the CA program were allowed to submit a CA program application by March 31, 2020. Medical technologies designated and listed as a B1 or B2 category between April 1, 2018, and March 31, 2020, can submit a CA program application until March 31, 2022. The process for reevaluation after granting CA eligibility is similar to that of C1 category application regardless of initial application categories.

Applications are permitted for the manufacturers or distributors of medical technologies only. Manufacturers who want to apply for the CA program must submit a plan for clinical data collection and evaluation. There are three formal requirements for the CA program, which are outlined in Table 2. In brief, eligibility criteria must be clearly outlined (Form 3-4) and submitted to the MHLW when the manufacturer submits the reimbursement dossier. If accepted, the applicant submits details of the evidence generated and collected at least every 2 years to the MHLW (Form 15). In the case of withdrawal, the manufacturer must provide the reason (Form 16).

**Table 2.** The Challenge Application (CA) Form and Requirements in Japan ^(43)^.

Form	Requirements
(Form 3-4) Eligibility of Challenge Application	• Manufacturers must submit this form to request eligibility for the Challenge Application program while they submit the dossier at an initial reimbursement application
	• Contents:
	-Eligibility of Challenge Application
	-Data collection and evaluation plan
(Form 15) Periodic report on Challenge Application	• Manufacturers must submit this form to the expert panel at a minimum of 2 years periodically
	• Contents:
	-Functions that manufacturers want to apply for Challenge Application, etc.
	-Status of data collection
(Form 16) Request for withdrawal	• If there is a legitimate reason, such as difficulty in collecting data, manufacturers can request withdrawal of eligibility of their Challenge Application
	• Once withdrawal is accepted, periodic reporting is unnecessary
	• Contents:
	-Reason for withdrawal and current status of data collection

## “Conditional Selective Benefit” Program in Korea

In Korea, there has been increasing demand on the coverage expansion of the national health insurance (NHI) system due to increased healthcare burden and high ratio of noncovered services and items. Since 2014, the government has implemented a CED system known as the “Selective Benefit (SB)” program to expand the health insurance coverage for four major diseases areas (cancer and heart, cerebrovascular, and rare diseases). SB mandates a high percentage of patient copayment rates ranging 30%, 50%, 80%, or 90% against normal patient copayment rate (5%, 10%, or 20%). The scope of coverage has been even further expanded with the introduction of the “Preliminary Benefit (PB)” program by the new government, which came into power in 2017. The new PB program is similar to the SB in terms of operation; however, the scope of items and services covered has been significantly expanded to noncovered services and items, with the exception of cosmetic and plastic surgery^(24)^.

The determination of eligible services and items for the SB program is made by considering clinical effectiveness, cost-effectiveness, and social demands on reimbursement coverage (Table 3). Technologies can be selected as CSB items if the following criteria are fulfilled: delivering technologies classified as high risk to patients, having a sophisticated procedural technique, or if supporting evidence for coverage determination are insufficient ^(25), (26)^. The MoHW decides the requirements for the eligible services and items for CSB in advance by official announcement. In addition, only healthcare providers that meet the specific requirements, including prerequisite clinical experience with the technology and possessing physician and facility qualification, are permitted to use the CSB services and items for their patients. In addition, they have an obligation to generate and submit the clinical data of their patients treated with the technologies ^(27)^.

**Table 3.** The Evaluation Criteria for the Selective Benefit Program in South Korea ^(35)^.

Aspects	Consideration elements
Clinical usefulness	①In case of proven clinical effectiveness:
	-If clinical effectiveness is proven equal or higher as an important clinical indicator when compared to alternative reimbursed items
	-If it is used for direct therapeutic purposes as an essential material in medical technologies
	-If increased diagnostic accuracy is proven and improved therapeutic outcome is expected in a diagnostic test
	②In case of proven improvement in medical process and it is expected to bring improved treatment outcomes:
	-If it is expected to bring improved treatment outcomes with improved medical processes, although there is not enough reasonable evidence when compared to alternative reimbursed items
	-If it is expected to bring improved treatment outcomes with proving improvement of convenience in medical technologies
	-If it is difficult to be expected to bring improved treatment outcomes, but the increase of the diagnostic accuracy is proven
	③In case of proven improvement in medical process, but it is not expected to bring improved treatment outcomes or the improvement in medical processes is not proven:
	-In case of medical technologies, if the improvement of convenience is proven, but it is not expected to bring improved treatment outcomes, or the improvement of convenience is not proven
	-In case of diagnostic tests, if the diagnostic accuracy is not proven
Cost-effectiveness	①In the case it is cost-effective:
	-If its effectiveness is similar or improved compared to alternative reimbursed items with the same costs or reduced costs
	②In the case it is not cost-effective or it is unclear:
	-If its effectiveness is similar or improved compared to alternative reimbursed items with high costs
	-Its effectiveness is low compared to alternative reimbursed items
Replaceability	①Irreplaceable cases:
	-If there are no reimbursed items available for patient
	-If there are no reimbursed items available for patients as an essential material for treatment
	②Replaceable case:
	-If there are reimbursed items that are available for patients
	-If it is implemented for support of existing items
	-If there are reimbursed items available for patients as an essential material for treatment
	-If it is used additionally according to the decision of users but not essential for the treatment
Social demand on reimbursement	①In case there is high social demand on reimbursement:
	-Considering the detailed assessment factors comprehensively, if there are high social interests and great power of influence for reimbursement
	②In case there is low social demand on reimbursement
	-Considering the detailed assessment factors comprehensively, if there is low social interest and great power of influence for the reimbursement

Under the CSB program, healthcare providers have an obligation to collect and report the clinical data to the Health Insurance Review and Assessment Service (HIRA). The HIRA, which has similar roles and responsibilities to the CMS in the USA, is accountable for operating the CSB program. Hospitals must obtain the approval from the MoHW and HIRA to participate in the CSB program and renew once a year to maintain its accreditation.

## Example of a Coverage with Evidence Development Program in Japan and Korea

### “Challenge Application” program in Japan: a pacemaker with enhanced functionality

A pacemaker with enhanced functionality, namely, the reactive anti-tachycardia pacing (rATP) algorithm, was submitted for the CA program in June 2019. The rATP algorithm delivers atrial anti-tachycardia pacing (ATP) to terminate an ongoing atrial fibrillation (AF) episode after a programmed interval or when the rhythm organizes and/or slows ^(28)^. The manufacturer submitted a C1 category application for the pacemaker in 2007 and obtained a new functional category with 5% utility premium for managed ventricular pacing mode. However, the rATP algorithm was not adequately assessed at the time because of a lack of appropriate clinical evidence. Five years later, a next generation of pacemaker, which was equipped with improved functionality of conditional magnetic resonance imaging (MRI) scan compatibility and rATP algorithm, was introduced. A C1 category application for the new version of pacemaker was submitted and obtained a new functional category with 5% improvement premium based on the MRI compatibility in 2012. While the conditional MRI scan compatibility was evaluated as it was considered a major improvement, the rATP algorithm was not taken into consideration again.

The new clinical evidence to indicate the feature of rATP algorithm was published in an international journal in 2014 ^(29)^. In addition, additional clinical evidence indicated that AF progression increases the risks of stroke and heart failure ^(30), (31), (32)^. After the introduction of the CA program, newly developed evidence was used to request a reevaluation of the rATP algorithm in 2019. The CA program was processed similar to that of a C1 category application, and the manufacturer showed the value of the enhanced functionality (rATP) that contributes to a reduction in the number of strokes and episodes of heart failure due to its suppression of AF progression. The MHLW acknowledged that the high probability of improvement in patient outcomes was due to the enhanced functionality of the pacemaker. The MHLW also created a new functional category and the provision of a 3% premium price in 2019. This was the first case of successful CA program implementation.

### “Conditional Selective Benefit” program in Korea: transcatheter aortic valve replacement

As of August 2020, three new medical technologies have been selected for the CSB program in Korea. These technologies include TAVR for the treatment of aortic stenosis (AS), percutaneous left atrial appendage occlusion for AF, and the next-generation sequencing technology-based genetic panel test. The TAVR, a minimally invasive technology treating symptomatic AS, was the first CSB item selected by the MoHW/HIRA. Healthcare providers submitted an application for a new procedure creation for TAVR; simultaneously, the manufacturers of TAVR technologies submitted a reimbursement application for the technology coverage and payment in 2013 after the new HTA approval was made in 2012 by the National Evidence-based Healthcare Collaborating Agency (HTA agency) ^(33)^. Two years after the submission, in June 2015, a positive CSB determination on TAVR was made after a thorough review of the clinical and economic benefit of TAVR to patients with AS ^(34)^.

Implementation method, records and management, and clinical data submission for CSB are roughly regulated by the law ^(35)^. In the case of TAVR, a national registry has been established to collect outcomes data and is currently in operation since 2017. An advisory group was established for the development and implementation of the TAVR registry protocol. The advisory group consisted of three clinical experts recommended by the Korean Society of Cardiology and Korean Society for Thoracic & Cardiovascular Surgery, and two experts in research methodology and statistics were added to form a total of eight members of the TAVR clinical and methodology advisory groups. Unlike foreign CED programs, key stakeholders, such as manufacturers and regulatory authorities (e.g., Ministry of Food and Drug Safety), are not involved in the governance of data collection.

After the initial decision on CED implementation, a reassessment is made every five year to determine whether the CSB needs to be maintained or transformed into formal reimbursement coverage benefit without any conditions for evidence collection. However, if conducting an earlier reassessment by considering the content, characteristics, and effects of the CSB services and items is deemed necessary, the reassessment cycle may change. To date, the reassessment for TAVR remains in progress.

The CSB program is limited to healthcare providers who have obtained approval from the MoHW/HIRA to participate by fulfilling the requirements specified in the program. As of December 2020, a total of 45 medical institutions have obtained approval for TAVR CSB implementation.

## Discussion

While the demand for the revaluation of safety, effectiveness, and cost-effectiveness of medical technology is increasing at the post-market stage (Table 4), CED is actively used globally to enable patient access to new medical technologies by ensuring reimbursement coverage during which time there is a commitment to generate further evidence. Both the CA and CSB programs are good examples of how countries in the Asia-Pacific region adopt and implement CED programs to their specific healthcare system needs. The CED programs in both countries are covered by the NHI system. Despite this similarity, the differences in regulatory frameworks and healthcare system operations make each CED program unique. The thorough review of these programs made it possible to identify commonalities, differences, and areas where improvement could be made (Table 5). The main disparities between the two programs include the responsibility of the applicant, nuances surrounding evidence generation, and expected administrative measures after reevaluation. In addition, premium prices are adjusted in Japan, while a copayment rate change or price adjustment can be made in Korea as a consequence of CED programs. While there are somewhat distinct characteristics in CED programs in both countries, the CED program provides a strong incentive for manufacturers by providing a favorable market access environment in terms of coverage and pricing determination at post-market stage.

**Table 4.** Comparison of Reevaluation Method of Medical Technology in Japan and Korea.

Reevaluation	South Korea	Japan
CED-driven	Reevaluation for CSB:	Reevaluation for CA:
	• Consider four components	• The process for reevaluation after granting CA eligibility is similar to that of C1 category application regardless of initial application categories
	-Clinical usefulness	
	-Cost-effectiveness	
	-Replaceability	
	-Social demand on reimbursement	
Non-CED-driven	Functional category reevaluation:	Not available
	• Review cost, effectiveness, and performance of listed items	
	• Adjust reimbursement price with the weighted average by applying claim volumes and prices	

CED, coverage with evidence development; CA, challenge application; CSB, conditional selective benefit

**Table 5.** The Comparison of Challenge Application (CA) and Conditional Selective Benefit (CSB).

Category	CA in Japan	CSB in South Korea
Authority in charge	MHLW	MoHW/HIRA
Applicant	Manufacturers	MoHW/HIRA
Target of evaluation	STMs	New procedures and medical technologies
Responsibility of evidence generation (data collection)	Manufacturers	Healthcare providers
Reevaluation period	Depending on protocol*	Designated by the MoHW/HIRA (usually 3-5 years)**
Patient financial burden (patient copayment)	Relatively low (e.g., 30%)	High (e.g., 80% patient copayment rates)
Expected administrative measures after reevaluation	Create new functional category and premium pricing	Relieve patient copayment rates or reduced prices

MHLW, Ministry of Health, Labour and Welfare; MoHW, Ministry of Health and Welfare; HIRA, Health Insurance Review and Assessment Service; STMs, Special Treatment Materials *Although reevaluation period varies depending on protocol, periodic report on CA program is needed to be submitted at least every 2 years. **According to the administration rule (standard for designation and implementation etc. of Selective Benefit), the designated period is determined a year after the designation of CSB.

It would be insightful for Japan and Korea to look for improvement areas by referring to the USA where CED programs have been effectively operating for several years. The operation and governance of CED programs in the USA is not exclusionary, with the active participation of multiple key stakeholders at specified timepoints in the CED pathway. An example of a CED program in the USA is the case of TAVR, which was established in May 2012 ^(36)^. This example provides insight and learning opportunities for countries that aim to introduce or improve CED programs in terms of transparency and collaborative commitment to generate evidences. In particular, when new medical technologies are introduced in the USA, local Medicare authorities determine whether to pay through local coverage determinations (LCDs) for a certain period of time; then, national coverage determinations (NCDs) are made. The CED program improves patient access to new medical technologies by directly incorporating them into NCDs without going through lengthy and cumbersome LCD process. In Japan and Korea, CED programs are similar in that they are applied nationwide; however, in fact, there is a big difference. In Korea, CSB is used under the restricted condition as divergence of SB program. As the patient copayment rate is quite high (80% for TAVR), it negatively impacts patient access to new medical technologies. In Japan, CED ensures patient access to new medical technologies, but lack of price premiums does not provide an incentive to companies to introduce new medical technologies. A premium price could be sought through post-market reevaluation after the development of robust local or international clinical evidence. Although the regulation for premium price determination currently exists, the ability to obtain a premium price for new and innovative medical technologies is currently very low (e.g., at best 3%-5% premium vs. listed conventional technologies), and this percentage has been decreasing since 2008 ^(37)^. The CED programs in Japan and Korea substantially differ from those in the USA, particularly in how they recognize the value of new medical technologies and also the presence of a patient copayment burden that unfavorably impacts patient access to new medical technologies.

Another important insight Japan and Korea can gain from the US CED programs is the speed and collaboration by which these programs are operated. Indeed, discussions on the development of a Transcatheter Valve Therapy (TVT) Registry began in July 2011, before the medical technologies used for TAVR were even approved by the US Food and Drug Administration (FDA) ^(26), (38)^. Multi-stakeholder discussions with government authorities meant that the registry was quickly established and incorporated variables that were to be used for the future evaluation of CED. In addition, an important characteristic of medical technologies, namely, the short product life cycle, was considered in the US CED program development ^(38)^. On the other hand, for CSB in Korea, the HIRA formed the advisory group to establish a systematic protocol for a TAVR registry in June 2017 ^(26)^, which was two years after the CSB program determination for TAVR was made. Unlike the case in the USA, the CSB program in Korea has been slow in communication because of lack of experience and preparedness. For the CA program in Japan, developing a registry is one option for applicants who want to generate evidence for reevaluation. If a registry is officially required, the evidence generation and collection plan is the responsibility of the applicant, and future reassessment by the MHLW is based on evidence collected by applicant. However, there have been no requirements for the establishment of a registry for CA to date.

Another key reason why the TAVR case in the USA is a successful example is the collaboration among multiple stakeholders. The Society of Thoracic Surgeons, American College of Cardiology, US FDA, and CMS participated in the creation of the TVT registry in partnership. Furthermore, they established the Stakeholder Advisory Committee to reflect multiple perspectives. The advisory committee includes a wide range of interest groups, including government, the medical technology industry, patient organizations, professional societies, etc^(38)^. In Korea, an advisory group for the TAVR registry was formed by inviting six clinical experts from the relevant specialty societies, and two methodological and statistical experts were consulted to establish the registry protocol ^(26)^. Unfortunately, representatives from medical technology manufacturers who have extensive knowledge and expertise in their technologies, patient populations, and expected outcomes were not included in the group. In Japan, CA is based on a protocol developed by the manufacturer, and the target of the CA program is only for STMs, not for procedures. Therefore, the leading role and participation of the manufacturers, which are familiar with medical technology related with STMs, are appropriate in CA initiation and process. While the medical technology industry requested the MHLW to extend the CA program for procedures in the C2 category, this is still under consideration. As the CA program has only recently been established, more discussions are needed to identify the advantages and disadvantages of this program. In addition, because of a lack of transparency on the internal decision-making processes of these programs, applicants have been unable to gain insight into what factors enable success or, more importantly, what could be rectified in the case of negative submissions. In the case of Japan, successful CA cases are available through the public notification of the MHLW, and failure cases are not publicized from the health authority. In the case of Korea, so far, a total of three technologies have been designated as CSB, and there are no results of success or failure yet.

Both countries have opportunities to include more active collaboration and participation in developing and governing CED programs with multiple stakeholders to ensure that the future success and value of these programs are realized. In the meantime, we need to be aware of the challenges of CED implementation, including the difficulties of withdrawal of the technologies because of safety or ethical issues. In particular, safety issue is very critical because the safety and effectiveness of the technologies subject to CED has not been established yet at the initial stage of CED implementation. As an example, the CMS provided coverage of lung volume reduction surgery for Medicare patients enrolled in the National Emphysema Treatment Trial over 7 years. However, it turned out that the surgery was associated with an increase in mortality, and the number of surgeries has significantly decreased ^(39), (40)^.

## Conclusion

Both Japan and Korea have been implementing CED programs since 2018 and 2014, respectively. Each country has established and operated its own unique approach to CED implementation, reflecting the contextual differences in their healthcare and policy environments. It is encouraging that both countries have introduced CED as a method to overcome the barrier of lack of robust clinical evidence in the early stages of medical technology introduction. The medical technology industry is open to CED because it provides an alternative mechanism for patient access to promising technologies that do not yet meet current evidence requirements. Despite the positive impact of CED programs in both countries, improvements in their governance and implementation should be made. The CED case in the USA provides insight on how to improve the CED operation in both countries. In particular, stakeholder engagement during the early stages of development and collaboration and partnership among the relevant stakeholders, including treating clinicians, governments/payers, patients/consumers, and medical technology industry, are required. The improvement of CED programs in Japan and Korea will enable patients in both countries to benefit by improved access to new and innovative medical technologies.

## Article Information

### Conflicts of Interest

All authors are the employees of Company Medtronic and stockholder.

### Author Contributions

Sang-Soo Lee and Jae-Eun Myung made substantial contributions to the conception and design of the work and drafted and critically revised it for important intellectual content. Yuji Tanaka, Hyunsook Choi, and Liesl Strachan revised the part of the manuscript regarding practical CED implementation in each country. Tomohiro Watanuki, Ji-Hyun Lee, and Hyojung Hwang helped edit and revised the manuscript. All of the authors approved the final manuscript.

### Approval by Institutional Review Board (IRB)

Not applicable
